# Prevalence of psychosis in black ethnic minorities in Britain: analysis based on three national surveys

**DOI:** 10.1007/s00127-014-0960-7

**Published:** 2014-09-11

**Authors:** Tarik Qassem, Paul Bebbington, Nicola Spiers, Sally McManus, Rachel Jenkins, Simon Dein

**Affiliations:** 1 Division of Psychiatry, Faculty of Brain Sciences, University College London, Charles Bell House, 67-73 Riding House Street, London, W1W 7EH UK; 2 Academic Unit of Social and Epidemiological Psychiatry, Department of Health Sciences, University of Leicester, 22-28 Princess Road West, Leicester, LE1 6TP UK; 3 NatCen Social Research, 35 Northampton Square, London, EC1V 0AX UK; 4 Department of Epidemiology and International Mental Health Policy, Institute of Psychiatry, King’s College London, London, UK; 5 Okasha Institute of Psychiatry, Ain Shams University, Cairo, Egypt; 6 Black Country Partnership NHS Foundation Trust, Edward Street Hospital, West Bromwich, West Midlands, UK; 7 Division of Mental Health and Wellbeing, University of Warwick, Coventry, UK

**Keywords:** Psychosis, Prevalence, National survey, Ethnicity, Mental health services

## Abstract

**Purpose:**

A considerable excess of psychosis in black ethnic minorities is apparent from clinical studies, in Britain, as in other developed economies with white majority populations. This excess is not so marked in population surveys. Equitable health service provision should be informed by the best estimates of the excess. We used national survey data to establish the difference in the prevalence of psychosis between black ethnic groups and the white majority in the British general population.

**Methods:**

Analysis of the combined datasets (*N* = 26,091) from the British national mental health surveys of 1993, 2000 and 2007. Cases of psychosis were determined either by the use of the Schedules for Clinical Assessment in Neuropsychiatry (SCAN), or from a combination of screening items. We controlled for sex, age, social class, unemployment, design features and other putative confounders, using a Disease Risk Score.

**Results:**

People from black ethnic minorities had an excess prevalence rate of psychosis compared with the white majority population. The OR, weighted for study design and response rate, was 2.72 (95 % CI 1.3–5.6, *p* = 0.002). This was marginally increased after controlling for potential confounders (OR = 2.90, 95 % CI 1.4–6.2, *p* = 0.006).

**Conclusions:**

The excess of psychosis in black ethnic minority groups was similar to that in two previous British community surveys, and less than that based on clinical studies. Even so it confirms a considerable need for increased mental health service resources in areas with high proportions of black ethnic minority inhabitants.

## Introduction

It is 100 years since Green [1] reported an increased rate of psychosis in black people in the USA. Higher rates of psychosis in ethnic minorities in general, and in black ethnic groups in particular, have been consistently replicated, and are almost universal in western industrialized countries. In a large English clinical study (AESOP), very high Incident Risk Ratios were found for schizophrenia in both African-Caribbeans (9.1) and Black Africans (5.8) [2]. In a meta-analysis of the incidence of psychosis in England, Kirkbride et al. [3] reported pooled risk ratios, compared with the white (British) population, of 5.6 (95 % CI 3.4–9.2) in the African-Caribbean population, and 4.7 (95 % CI 3.3–6.8) in the black African population. In contrast, South Asian groups had an RR of 2.4.

These results have considerable public health implications, but the locations of the clinical studies have tended to be circumscribed. They would thus be amplified by epidemiological surveys of whole populations. However, these too have disadvantages: both their sample size and their geographical spread may be limited. Sample size is particularly challenging in the case of less common disorders like psychosis.

In the UK, there have been few community-based studies of the effects of ethnicity on rates of psychosis. Two have examined the African-Caribbean minority population (but not black Africans). In contrast to the clinical studies, the Fourth National Survey of Ethnic Minorities [4] reported a relatively small excess of psychosis in African-Caribbeans. The prevalence, at 1.4 %, was only 1.75 times the white rate, with the excess entirely restricted to female participants. The EMPIRIC survey [5] calculated a prevalence in the white subgroup of 0.8 %, while that in the African-Caribbeans was 1.6 %. There was little gender difference in the African-Caribbean group.

The existence of a high prevalence of psychosis in black ethnic minorities has several implications. It might be related to their particular social, cultural and religious characteristics. However, in Britain, although there are marked cultural differences between black Caribbean and black African minorities, both have increased rates of psychosis in clinical studies. Despite these cultural differences, black groups share the experience of discrimination and consequent social disadvantage because they share distinguishing physical features. This may be the main driver of the high rates of psychosis. Racist attacks and perceived employer racism are associated with increased rates of psychosis in black ethnic minorities [6]. Racism also appears to reduce willingness to comply with prescribed medication, which might thereby increase admission rates [7]. The particular causes may shape the form of appropriate service provision. However, whatever the mechanisms, the increased prevalence bears on the equitable funding of services: areas with greater numbers of people from these ethnic minorities will merit higher levels of funding.

The British National Psychiatric Morbidity Surveys provide a further opportunity for estimating the scale of the excess prevalence and its potential public health consequences [8]. There have been three household surveys, in 1993, 2000 and 2007. Even so, the size of the individual surveys is on the small side for detailed studies of psychosis in relation to variables such as ethnic groupings. In this paper, we have increased statistical power by amalgamating the data from all three, giving a combined sample size of over 26,000. It was possible to do this because central aspects of the design and methods of the individual surveys remained the same.

The focus of the current paper is on the relationship between membership of the amalgamated black ethnic groups and the prevalence of psychosis. It had the following aims:

1. To establish the prevalence of psychosis in people from black ethnic groups relative to the white British population.
2. To control for putative confounders, particularly design features, age, social class and employment status.
3. To estimate the impact of variations in the proportion of people from black ethnic minorities on the prevalence of psychosis.

## Methods

### Setting and design

The 1993 and 2000 surveys covered all of Great Britain except the Highlands and Islands of Scotland, while the 2007 survey covered only England. Full details of the survey methods can be found elsewhere [9–11]. The targeted age range was extended in successive surveys, being 16–64 in 1993, 16–74 in 2000 and 16 and upwards in 2007. The sample sizes of each survey were designed to have the statistical power required for estimating the prevalence of less common disorders (0.5–1.0 %) by age, sex and region. The number of individuals who successfully completed phase 1 interviews in the three surveys was 26,091 (10,108, 8,580, and 7,403, respectively). In all three surveys, the sampling frame was the Small User Postcode Address File. Adults living in private households were sampled using population-based multi-phase probability sampling. Experienced survey interviewers identified private households containing at least one person. They used the Kish grid method [12] to select at random one person in each household, ensuring that all eligible household members had the same chance of being selected. While some changes and improvements were made in successive surveys, the emphasis was on retaining a majority of the instruments used, to allow comparison. Stratification of primary sampling units by region and socioeconomic characteristics was more fine grained in 2007 than in 2000 and 1993, but in each case data could be weighted to take account of survey design and non-response, to render the results representative of the household population in the chosen age range. It was possible to apply these survey weights to individuals in the combined dataset.

### Phase 1 and phase 2 interviews

Phase 1 interviews were carried out by the survey interviewers, using a detailed questionnaire which established socio-demographic characteristics, as well as covering a range of other topics. Screening procedures were applied to determine eligibility for the phase 2 interviews, which were carried out by clinically trained research interviewers.

### Ethnic grouping

The participants were asked to allocate themselves to an ethnic group, using the same categories as the most recent census. Because of the relatively small numbers in most groups, categories were collapsed into larger groupings: the white ethnic majority, which included all white participants (whatever their country of origin), black ethnic groups (covering black Caribbean, black African, black other, and mixed white/black), and a residual, ‘Other’, group. The white group comprised 93.2 % of the population, the black ethnic groups 2.1 %, and the ‘Other’ group (which included South Asians and Chinese) 4.0 %. Ethnicity data were missing for 0.7 % of participants.

In the analyses that follow, we used only data on individuals from the white and black ethnic groups, except where we calculated the effect of the proportion of black ethnic individuals in the total population on the prevalence of psychosis, when we amalgamated the white ethnic majority and “Other” groups.

### Social class

Social class was classified according to the Registrar General’s classification, and divided into three groups: social classes I and II, social class III, and social classes IV and V (this last group also included members of the armed forces). In the 1993 survey, a married or cohabiting woman was only classified according to her own occupation if her partner was not currently working. In subsequent surveys, married or cohabiting women were always classified on the basis of their own occupation. It was not possible to recreate the later procedure in relation to the 1993 data.

### Employment status

We categorized employment status as employed, unemployed and economically inactive. Unemployment was twice as common in the black ethnic minority groups as in their white counterparts, whereas the economically inactive proportion did not differ.

### Identifying a history of illicit psychoactive drug use

Participants were asked about their lifetime usage of cannabis, stimulants (cocaine, amphetamines) and psychedelic drugs (LSD, etc.), using questions based on the Diagnostic Interview Schedule [13].

### Identifying psychotic disorders

Our analysis was based on criteria for psychosis relating to the past year. In each survey, participants were screened during phase 1 for possible psychosis, a process that included the Psychosis Screening Questionnaire (PSQ) [14]. They were invited for a phase-two assessment of possible psychosis if they met one or more of the following criteria:

- Currently on anti-psychotic medication.
- An in-patient stay for a mental or emotional problem in the past 3 months, or admission to a hospital or ward specializing in mental health problems at any time.
- A positive response to question 5a in the PSQ. This relates to auditory hallucinations.
- A self-reported diagnosis of psychotic disorder or of symptoms suggestive of it.

For the purpose of analysis, participants not meeting any of these criteria were assumed not to have psychosis.

Of those invited for a second phase interview in each of the three surveys, 63.2, 61.6, and 74.2 % attended. For people interviewed in phase 2, the diagnosis of psychosis was based on the SCAN system, a semi-structured clinical interview that, with its attendant algorithm, provides ICD-10 diagnoses of psychotic disorder [15]. In view of the expected low prevalence of psychotic disorders, a single category was created corresponding to diagnoses of schizophrenia, schizoaffective disorder, and affective psychosis. In the analyses presented here, we followed the procedure in the 2000 and 2007 survey reports of establishing a measure of “probable psychosis”, and applied it retrospectively to the 1993 data. This category included all cases identified through SCAN interviews, together with some participants who were not interviewed with SCAN. The latter were chosen because they met *at least two* of the phase 1 psychosis screening criteria listed above. A detailed description of the rationale for this procedure is set out in the Technical Report of the 2000 survey (pp 31–33) [16]. There were no differences between cases identified by SCAN interviews (*N* = 85) and those identified by the application of the algorithm (*N* = 81) in age, sex, ethnicity, educational qualifications, employment status or social class. The probable psychosis measure was used in the official reports of the surveys [9–11] and has been adopted consistently in investigations of psychosis based on these datasets [17–20].

### Analyses

Data from each survey were weighted to allow for design and response rates: this procedure was necessarily complicated, and is described in full detail in the relevant reports [9–11]. To allow for weighting, the data were analyzed using the Statistical Package for the Social Sciences (version 18 for Windows). Binary logistic regression analyses were used to calculate the odds ratios (ORs) and 95 % confidence intervals (CIs) contrasting the black ethnic groups and the white group in relation to the presence of psychosis. Our analyses also involved evaluation of the effect of controlling for a number of other variables, albeit constrained both by the danger of over-control in the face of limited numbers of cases, and the fact that some variables were lost to us by successive changes in the way they were coded (for instance, life events and material affluence). We first chose to control for two indicators of design differences: residence in England and the year of the survey. Subsequently, further control variables were selected on the grounds that they might be expected to modify the relationship between ethnicity and psychosis. Adjusting for age was important, as there were differences in the age-bands covered by the surveys. Age differences between ethnic groups might also lead to a degree of spuriousness in the relationship between ethnicity and psychosis, although this would be a more likely consequence in relation to incidence than (as here) to prevalence. We also identified social class, employment and psychoactive drug use as potential confounders.

To address the problem arising from the large number of potential confounders and the relatively small number of patients from black ethnic minorities with psychosis, we adopted the technique of controlling for a Disease Risk Score (DRS) in establishing the odds ratio relating black ethnic minority status to psychosis. The DRS estimates the probability or rate of disease occurring as a function of multiple covariates in situations where many are likely to apply [21, 22]. It permits entry of variables considered to be putative confounders, even when their association with psychosis is not statistically significant. The objective is to build a score that summarizes as much information from confounders as the dataset will permit. However, the scope for this is not unlimited, as only 166 cases of psychosis were identified. Given a rule of ten events per parameter [23], the complexity of the model should thus be restricted to a maximum of 16 parameters.

Our calculation of the DRS was based on an initial logistic regression of the following risk factors: ethnic grouping, sex, age, educational qualifications, social class, unemployment, and the use of cannabis, stimulants and psychedelics, together with year of the survey, and whether participants lived in England or not. The resulting ORs were then multiplied by the individual covariate values of the variables entered into the model, with the exception of ethnicity. The sum of these products gave the participant-specific DRS, which was then used to control for confounding in a separate regression model. In this model, the independent variables comprised only ethnicity and the DRS (as a continuous predictor).

#### Population impact analysis

To calculate the effect of different proportions of people from black ethnic minorities in local populations on the prevalence of probable psychosis, we used the following formula:

$$\begin{aligned} {\text{prevalence}} & = \left( {{\text{BEM prevalence}} \times {\text{BEM population proportion}}} \right) \\ & \quad + ({\text{nonBEM prevalence}} \times (1 - {\text{BEM population proportion}})) \\ \end{aligned}$$

## Results

Out of 24,318 white participants, 156 had probable psychosis, giving a prevalence, after weighting, of 5.2 per 1,000. In contrast, of 549 individuals of black ethnic minority background, 10 had probable psychosis, a weighted prevalence of 14.5 per 1,000 (OR 2.72, 95 % CI 1.31–5.63, *p* = 0.002).

There was no statistical difference in sex distribution between black ethnic minority and white participants. However, the former were significantly more likely to be under the age of 45 than their white counterparts (OR 1.80, 95 % CI 1.50–2.16, *p* < 0.001), and they were significantly over-represented in the lower class group (skilled manual, partly skilled, and unskilled occupations, and those who had never worked; OR 1.44, 95 % CI 1.20–1.72, *p* < 0.003). 9.3 % of the black groups were unemployed, compared to 4.9 % of the white participants, resulting in a significant OR of 2.4 (95 CI 1.7–3.2, *p* < 0.001). There was no difference in the use of cannabis between the white ethnic majority and black ethnic minorities (OR 1.01, 95 % CI 0.81–1.26, *p* = 0.922). Black ethnic minority participants reported significantly *lower* rates of use of stimulants (OR 0.57, 95 % CI 0.37–0.87, *p* = 0.009) and of psychedelic drugs (OR 0.51, 95 % CI 0.30–0.87, *p* = 0.013).

The regression model used in calculating the DRS is shown in Table 1. It should be noted that, in this multivariate analysis, differences in the design of the surveys (as reflected in the year of the survey and the country of residence) had no effect on the prevalence of psychosis. Nor did sex, educational qualifications, or the use of cannabis, stimulants, or psychedelic drugs. The variables associated with psychosis were age, ethnic grouping, social class (specifically membership of social classes IV and V), and employment status (especially being economically inactive).

**Table 1:** Logistic regression for probable psychosis used in calculating the Disease Risk Score

		Significance	OR	95 % CI for OR	
				Lower	Upper
Year of survey (reference: 2007)					
2000		0.23	1.34	0.83	2.16
1993		0.33	1.27	0.79	2.03
Location (reference: England)					
Wales or Scotland		0.83	1.07	0.61	1.86
Ethnic grouping (reference: white majority)					
Black groups		0.008	2.80	1.30	6.03
Sex (reference: male)					
Female		0.20	0.79	0.54	1.13
Age (reference: 45 years or more)					
Less than 45 years		0.03	1.61	1.04	2.50
Social class (reference: classes I and II)					
Class III		0.28	1.30	0.81	2.08
Classes IV and V		0.021	1.82	1.09	3.01
Educational qualifications (reference: any educational qualification)					
None		0.93	1.02	0.67	1.54
Employment status (reference: employed or economically inactive)					
Not employed		0.038	2.19	1.05	4.60
Economically inactive		0.0001	5.83	3.85	8.84
Cannabis use (reference: no usage)					
Use reported		0.24	1.37	0.81	2.34
Stimulant use (reference: no usage)					
Use reported		0.25	1.55	0.74	3.22
Use of psychedelic drugs (reference: no usage)					
Use reported		0.19	1.67	0.78	3.58

We then used the Disease Risk Score, constructed as described above, to analyze the combined effect of possible confounders (irrespective of whether they were significantly associated with ethnic status individually) on the link between ethnicity and psychosis. The analysis based on controlling for the DRS produced a marginal increase in the odds ratio (from 2.72 to 2.90) (see Table 2). It should be noted that the DRS included variables that reflect levels of social disadvantage (social class, educational status, employment status).

**Table 2:** Odds of probable psychosis and overall effect of controlling for potential confounders as expressed in Disease Risk Score (DRS)

DRS^a^	OR = 1.45 (1.34–1.57)	*P* < 0.0001
Belonging to BEM	OR = 2.90 (1.4–6.2)	*P* = 0.006

^a^DRS calculated from a model including year and location of survey, age, gender, educational qualification, social class, unemployment, and use of cannabis, stimulants and psychedelic drugs

**Fig. 1:**
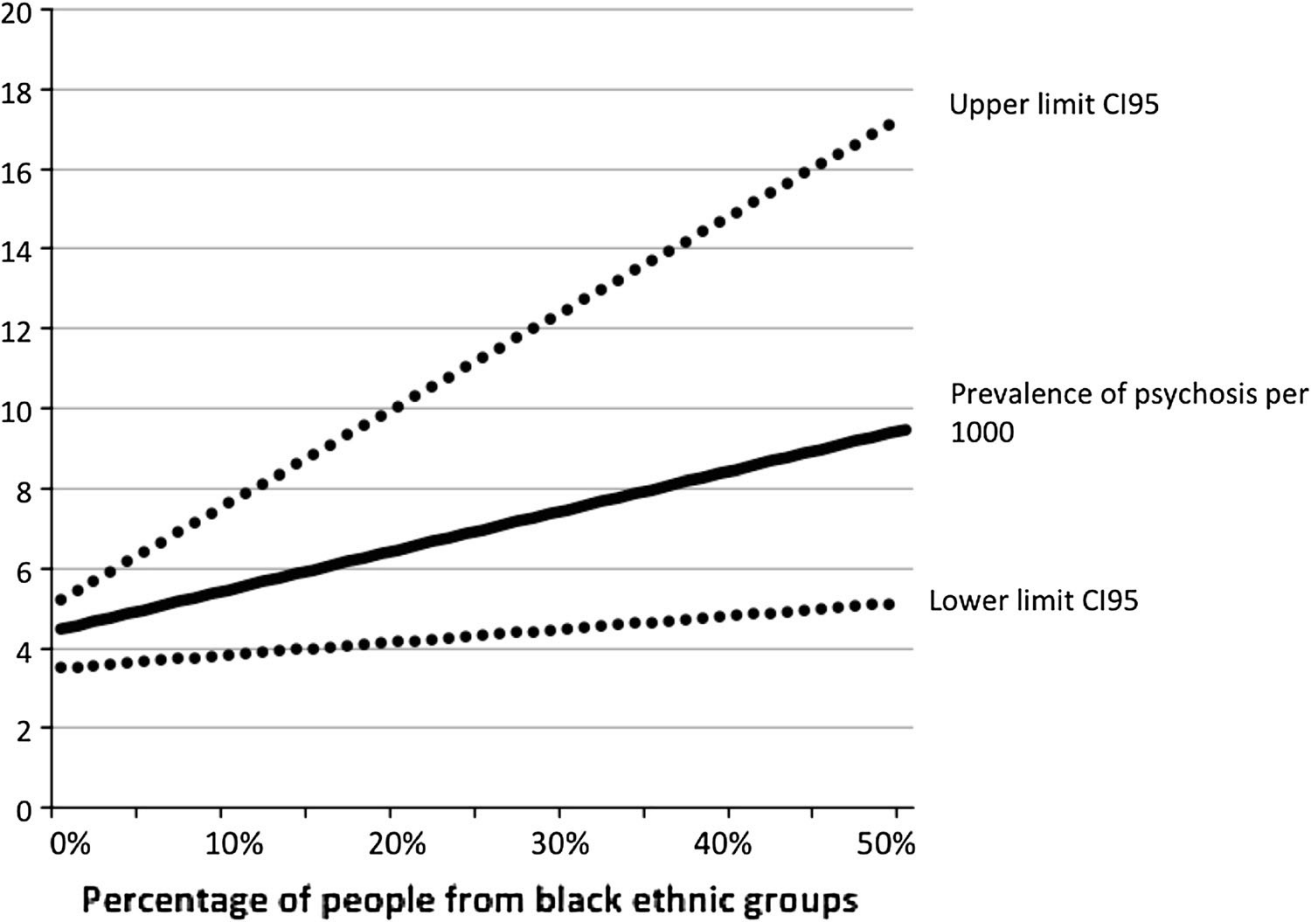
The ethnic composition of the population and the putative prevalence of psychosis

People from black ethnic groups comprised 2.2 % of the combined survey samples. Given relative odds of 2.90 for a diagnosis of psychosis in comparison to white participants, the presence of a black ethnic population of this size would increase the prevalence of psychosis by 3.3 %. The overall effect on the requirements for appropriate services is thus small. However, in some super-output census areas, particularly in London, this rises to over 40 %. An area with 40 % of inhabitants from black minorities would, on the basis of our figures, have a 76 % increase in demand for services. The changing relationship between population composition and prevalence is shown in Fig. 1. The relationship is linear because the prevalence of psychosis in both the black ethnic minority population and in the rest of the population is assumed to be unchanged as the black ethnic minority proportion increases. In practice, if we accept the putative effects of ethnic density, the relationship is more likely to follow a convex curve.

## Discussion

This study uses the amalgamated data from three national household psychiatric surveys carried out in Britain in 1993, 2000 and 2007. It provides the largest such dataset from community-based studies in the UK, although other, smaller, surveys have used booster samples from minority groups to improve statistical power. The weighted prevalence of psychosis per 1,000 in the white population was 4.4, while that in the black ethnic minority population was 14.2. The adjusted relative odds of developing psychosis in black survey participants relative to the white majority population were 2.9, with confidence limits between 1.4 and 6.2 (*p* = 0.006).

This result was robust in the face of our incorporation of potential confounders in analysis. There was no evidence of confounding by sex or age, or by membership of individual surveys spread over a 14-year period. There was no difference between black ethnic minority and white participants in their use of cannabis, while the use of psychedelics and stimulants was significantly more prevalent in the latter

Class, education and employment status are measures of disadvantage. There was indeed a significant difference in social class between white participants and those from black ethnic groups: the latter were over represented in the lower social classes, especially in unskilled occupations. They also had higher rates of unemployment but were less likely to be economically inactive. However, the raised relative odds of probable psychosis in the black ethnic groups hardly changed when we controlled in logistic regression for the calculated Disease Risk Score, which included social class and employment status. Ideally, we would have liked to control for victimization events, which are strongly related to psychosis [17], and may be unevenly distributed between black and white members of the population. However, these were not recorded in the 1993 survey.

While this community-based analysis suggests a clear excess of psychosis in black ethnic groups, the excess is not as great as that reported in clinical studies. It is of interest that the two other community surveys in the UK have reported similar relatively small excesses [4, 5]. This difference from the clinical studies could have arisen by chance, as the 95 % confidence limits of the results from the two types of studies overlap. However, the consistency of the discrepancy across the community surveys might indicate a substantive difference.

Although the community surveys differ in sampling strategy and in the identification of psychosis, the differences between them are not marked. The Fourth National Survey of Ethnic Minorities [4] included 2,867 white participants and a boost sample of 427 African-Caribbeans. The diagnosis of psychosis was based either on the second phase interview with the ninth edition of the Present State Examination [24], or reports of taking anti-psychotic medication. The EMPIRIC survey [5] used a similar approach to sampling, with a boosted sample of ethnic minorities. In all, 837 white and 694 African-Caribbean participants were enlisted. The prevalence of psychotic disorder was calculated from an algorithm using PSQ scores [14] to estimate the likelihood of psychosis.

Our results provide an estimate of the effect of the black ethnic minority population on the appropriate allocation of resource to psychiatric services, and indicate that, even with relatively small excesses in prevalence, the impact may be appreciable in areas with large ethnic minority populations. Even so, we do not know whether people with psychosis from black ethnic groups will have greater requirements from services than those from the majority population. If they do, it would be a potential explanation for the discrepancies between community and clinical studies. Moreover, it would add to the increased requirement for services in areas serving substantial black ethnic minority populations. Given that around 27 % of service costs for schizophrenia are in-patient costs [25], disproportionate bed occupancy would further increase the need to boost funding. Thus, the population impact analysis may provide usable guidance on the supplementary funding required if equity is to be maintained.

### Limitations

Even with the large overall sample size, the number of people with psychosis among black participants was inevitably small. We were unable to compare the prevalence of psychosis in the separate black African-Caribbean and black African groups, due to the small numbers of individuals with psychosis. The response rates from individuals with active psychosis, or those from deprived backgrounds are unlikely to be as good as for the rest of the population, and this may lead to unpredictable distortions in the case sample. Further distortion might accrue from the impact of cultural issues and consciousness of stigmatization on responses to the questionnaires. Non-English speakers were excluded from the survey: however, few of these would have been from black ethnic groups at the time of these surveys. Our overall 1 year prevalence of psychosis was, as would be expected, somewhat less than lifetime rates, but is consistent with quoted values: in their systematic review, McGrath et al. [26] cite figures for the lifetime morbid risk of narrowly defined schizophrenia of 0.72 %. Perälä et al. [27] reported a lifetime prevalence of all forms of non-affective psychosis of 1.44 %. However, this would have been inflated by the fact that their sample was aged over 30.

Finally we were unable to control for ethnic density, the proportion of given ethnic groups living in the total population of a given area. Areas of low ethnic density are associated with increased rates of psychiatric disorder in people from ethnic minorities. Living in areas of low ethnic density is likely to increase exposure to isolation, discrimination, racism and disadvantage. It has a particularly strong effect on psychosis in African-Caribbean groups [28]. This would affect our calculation of population impact (the straight line in Fig. 1 would have become a convex curve, with some reduction in the service cost implications of increasing proportions from black ethnic groups). We could not control for it in analysis, as information about the area of residence of participants is embargoed in the archived datasets on grounds of confidentiality. Nor could the published literature provide values for the effects of ethnic density in a form that would allow us to estimate its effect on the curve.
